# Diagnostic accuracy of dual-phase ^18^F-FP-CIT PET imaging for detection and differential diagnosis of Parkinsonism

**DOI:** 10.1038/s41598-021-94040-8

**Published:** 2021-07-22

**Authors:** Minyoung Oh, Narae Lee, Chanwoo Kim, Hye Joo Son, Changhwan Sung, Seung Jun Oh, Sang Ju Lee, Sun Ju Chung, Chong Sik Lee, Jae Seung Kim

**Affiliations:** 1 Department of Nuclear Medicine, Asan Medical Center, University of Ulsan College of Medicine, 88, Olympic-ro 43-gil, Songpa-gu, Seoul, 05505 Korea; 2 Department of Nuclear Medicine, Wonju Severance Christian Hospital, Yonsei University Wonju College of Medicine, Wonju, Korea; 3 Department of Neurology, Asan Medical Center, College of Medicine, University of Ulsan, Seoul, Korea; 4 Department of Nuclear Medicine, Kyung Hee University School of Medicine, Kyung Hee University Hospital At Gangdong, Seoul, Korea

**Keywords:** Neuroscience, Neurology

## Abstract

Delayed phase ^18^F-FP-CIT PET (dCIT) can assess the striatal dopamine transporter binding to detect degenerative parkinsonism (DP). Early phase ^18^F-FP-CIT (eCIT) can assess the regional brain activity for differential diagnosis among parkinsonism similar with ^18^F-FDG PET. We evaluated the diagnostic performance of dual phase ^18^F-FP-CIT PET (dual CIT) and ^18^F-FDG PET compared with clinical diagnosis in 141 subjects [36 with idiopathic Parkinson’s disease (IPD), 77 with multiple system atrophy (MSA), 18 with progressive supranuclear palsy (PSP), and 10 with non-DP)]. Visual assessment of eCIT, dCIT, dual CIT, ^18^F-FDG and ^18^F-FDG PET with dCIT was in agreement with the clinical diagnosis in 61.7%, 69.5%, 95.7%, 81.6%, and 97.2% of cases, respectively. ECIT showed about 90% concordance with non-DP and MSA, and 8.3% and 27.8% with IPD and PSP, respectively. DCIT showed ≥ 88% concordance with non-DP, IPD, and PSP, and 49.4% concordance with MSA. Dual CIT showed ≥ 90% concordance in all groups. ^18^F-FDG PET showed ≥ 90% concordance with non-DP, MSA, and PSP, but only 33.3% concordance with IPD. The combination of ^18^F-FDG and dCIT yielded ≥ 90% concordance in all groups. Dual CIT may represent a powerful alternative to the combination of ^18^F-FDG PET and dCIT for differential diagnosis of parkinsonian disorders.

## Introduction

Parkinsonism is a clinical syndrome characterized by bradykinesia, rigidity, rest tremors, and postural instability. Idiopathic Parkinson's disease (IPD) is the most common cause of parkinsonism and is associated with progressive neuronal loss of the substantia nigra and other brain structures^1,2^. The differential diagnosis of IPD versus other forms of parkinsonism is important for the treatment and prognosis. However, this is challenging, especially early in the disease when signs and symptoms of different forms of parkinsonism have greater overlap, and the error rates in clinicopathological series are as high as 20%^3^. Such signs and symptoms can also be present in patients with degenerative parkinsonism (DP), including multiple system atrophy (MSA) and progressive supranuclear palsy (PSP), non-degenerative parkinsonism (non-DP), including essential tremor (ET), or other secondary parkinsonism, including drug-induced parkinsonism (DIP)^4^.

Presynaptic striatal dopamine transporter (DAT) density has been shown to correlate with the density of dopaminergic neurons. DAT imaging has high sensitivity and specificity for discriminating DP from non-DP^5^, and is approved for clinical use in several countries^6^. Among them, N-(3-fluoropropyl)-2β-carboxymethoxy-3β-(4-iodophenyl) nortropane (^18^F-FP-CIT) is a PET tracer with demonstrated ability for the detection and differentiation of DP^7–11^, based on its higher resolution and specific binding ratio compared to ^123^I-FP-CIT SPECT^12^.

In addition to DAT tracers, ^18^F-fluorodeoxyglucose (^18^F-FDG) PET is a sensitive marker that has been used for the differentiation of DP^13–15^, and has yielded concordant or superior results compared to striatal dopamine D2/D3 receptor (D2R) imaging^16^. Moreover, combination of DAT and ^18^F-FDG or perfusion imaging has shown accurate differentiation between the common forms of DP^17–19^. Despite this, several clinical drawbacks, including costs, radiation exposure, reimbursement issues, and the need to visit hospital at least twice, limits the utilization of dual tracer imaging in clinical settings.

It has previously shown that early phase imaging of PET tracers with high lipophilicity, including ^18^F-FP-CIT, is a potential surrogate biomarker of overall functional brain activity, and has the potential to be used as a substitute for ^18^F-FDG PET^20–24^. Therefore, the goal of the present work was to study the diagnostic performance of dual phase ^18^F-FP-CIT PET imaging (early and delayed) for the differential diagnosis of parkinsonism compared to ^18^F-FDG PET alone or in combination with ^18^F-FP-CIT PET.

## Methods

### Subjects

Subjects who underwent dual-phase ^18^F-FP-CIT and ^18^F-FDG PET examination for work up for parkinsonism within 5 years of symptom onset from September 2008 to August 2016 were included. All patients were assessed by a neurologist specializing in movement disorders. Among patients with parkinsonism, the diagnosis of IPD was based on the UK Parkinson’s Disease Society Brain Bank Clinical Diagnostic Criteria^25^. Patients with clinically probable MSA-P, MSA-C, or PSP were enrolled based on current diagnostic criteria^26,27^. Their structural MRI was also assessed in order to evaluate other neurological disease, and those who had infarction in the striatum were excluded. The scanning interval between ^18^F-FP-CIT PET and ^18^F-FDG PET was less than 1 year. The Institutional Review Board (IRB) in the Asan Medical Center(IRB no. 2020–0442) approved this study protocol and waived informed consent from patients due to the retrospective nature of the study.

### ^18^F-FP-CIT PET/CT imaging

^18^F-FP-CIT PET was performed using a Biograph 40 TruePoint PET/CT camera (Siemens Medical Systems, USA), which provides an in-plane spatial resolution of 2.0 mm full width at half maximum at the center of the field of view. Images were acquired from 0 to 10 min for early phase and 180–190 min for delayed phase after intravenous injection of ^18^F-FP-CIT (185 MBq). Emission PET data were acquired in the 3-dimensional mode after brain CT, which was performed in the spiral mode at 120 kVp and 380 mA (reference standard) using the CARE Dose 4D program. ^18^F-FP-CIT PET images were reconstructed from CT data for attenuation correction using the TrueX algorithm and an all-pass filter with a 336 × 336 matrix.

### ^18^F-FDG PET/CT imaging

The ^18^F-FDG PET scans were acquired using an ECAT HR + scanner (CTI-Siemens, Knoxville, Tennessee, USA) in 30 subjects, and using a Discovery 690 scanner (GE healthcare, Waukesha, Wisconsin, USA) in 111 subjects. All subjects fasted for a minimum of 6 h before ^18^F-FDG PET imaging.

In patients who were studied by the ECAT HR + scanner, transmission scans of 5 min using a rotating pin source of germanium-68 for attenuation correction, and emission scans of 15 min were acquired 40 min after intravenous injection of 370 MBq of ^18^F-FDG. In-plane and axial resolutions of the scanner were 4.3 mm and 8.3 mm full width at half maximum (FWHM), respectively. Following acquisition, ^18^F-FDG PET images were reconstructed using a Gaussian filter (FWHM, 2 mm) and displayed in a 128 × 128 matrix (pixel size, 1.72 × 1.72 mm with a slice thickness of 2.43 mm). Emission images were reconstructed with ordered subset expectation maximization (OSEM) using 16 subsets and 6 iterations.

In patients studied by the Discovery 690 scanner, computed tomography data for attenuation correction were acquired first, followed by three-dimensional ^18^F-FDG PET image acquisition. In-plane and axial resolutions of the scanner were 4.9 mm and 5.6 mm FWHM, respectively. ^18^F-FDG PET images were reconstructed using a Gaussian filter (FWHM, 2 mm) and displayed in a 256 × 256 matrix (pixel size, 0.98 × 0.98 mm with a slice thickness of 3.27 mm). Emission images were reconstructed with OSEM using 24 subsets and 2 iterations.

### Visual assessment

**Figure 1:**
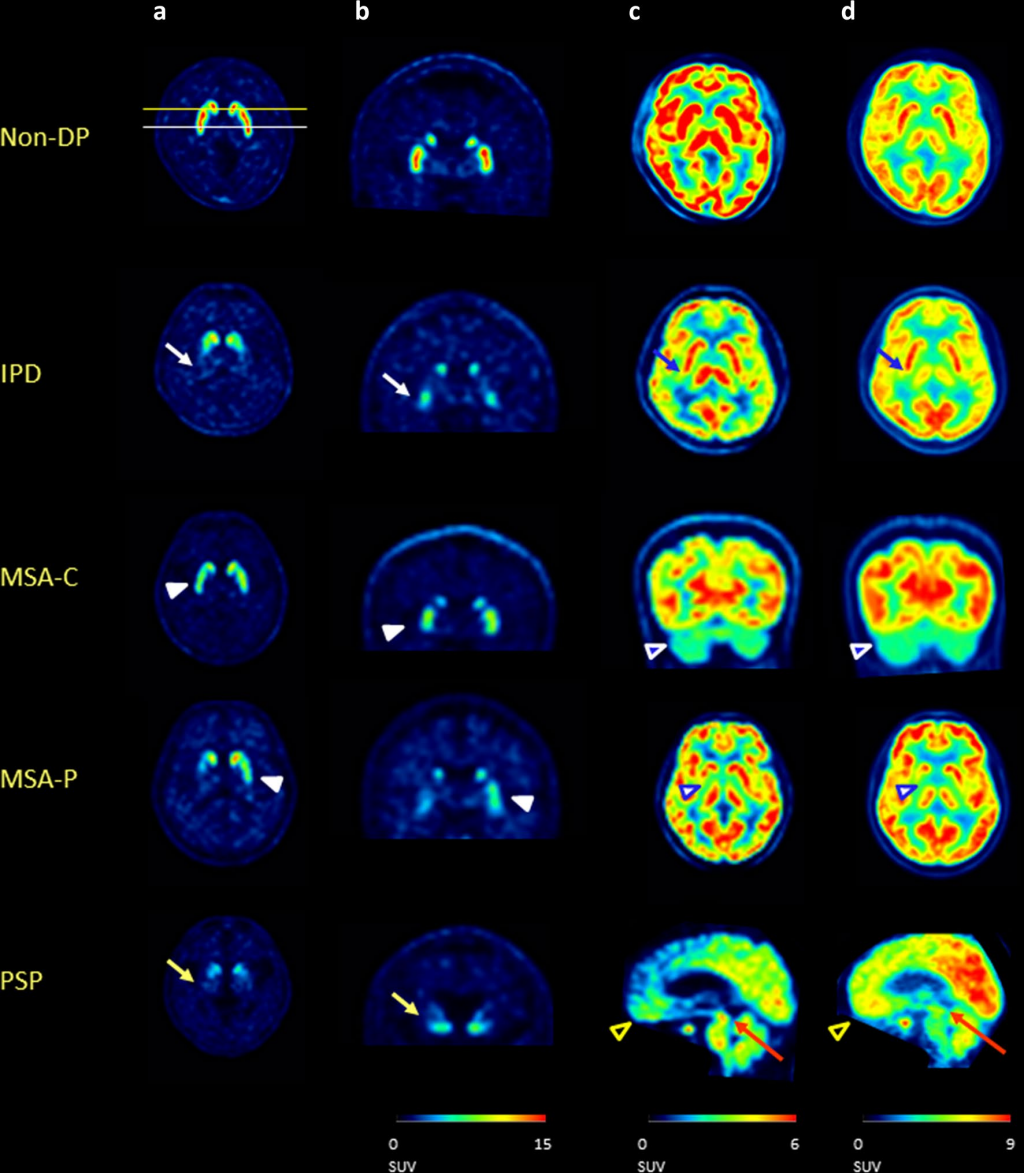
Examples of axial (**a**) and coronal slice (**b**) of delayed phase ^18^F-FP-CIT PET, early phase ^18^F-FP-CIT PET (**c**), and ^18^F-FDG PET (**d**) of patients with non-degenerative parkinsonism (1st row), IPD (2nd row), MSA-C (3rd row), MSA-P (4th row), and PSP (5th row). The patient with IPD (2nd row) showed decreased DAT binding of the bilateral dorsal posterior putamen, with relative sparing of the ventral putamen (white arrow) on delayed phase ^18^F-FP-CIT PET, and normal striatal metabolism or hypermetabolism in the bilateral dorsolateral putamen on early phase ^18^F-FP-CIT PET and ^18^F-FDG -PET (blue arrow). The patient with MSA-C and MSA-P (3rd and 4th rows), shows DAT loss not only in the posterior putamen, but also in the ventral putamen (white arrow head) on delayed phase ^18^F-FP-CIT PET. On early phase ^18^F-FP-CIT PET and ^18^F-FDG -PET, MSA showed hypometabolism in the cerebellar cortex (blue arrow head with white outline) and/or hypometabolism in the basal ganglia (blue arrow head with white outline. The patient with PSP showed extensive DAT loss on both the putamen and caudate nuclei on delayed phase ^18^F-FP-CIT PET (yellow arrow), which was characterized by hypometabolism in the medial frontal cortices (black arrow head with yellow outline) and midbrain (red arrow) on early phase ^18^F-FP-CIT PET and ^18^F-FDG PET. All PET images were processed using PMOD 4.0 (PMOD Technologies Ltd., Zurich, Switzerland).

All PET images were visually assessed by a panel of two independent observers, who were board-certified nuclear medicine physicians with 20 and 5 years of clinical experience in neuro-nuclear medicine for each. Any discrepancies were resolved by a third reviewer. They were blinded to all clinical and diagnostic information. The visual interpretation criteria for the diagnosis of parkinsonism for delayed phase ^18^F-FP-CIT PET was based on the pattern of DAT loss in the striatum as described previously, and classified as non-degenerative parkinsonism (non-DP), IPD, MSA, or PSP^8^. Representative PET images are shown in Fig. 1. Briefly, the pattern of non-DP was defined as symmetric and even DAT binding without focal deficit in the bilateral striatum. For IPD, decreased DAT binding of the bilateral dorsal posterior putamen was observed, with relative sparing of the ventral putamen. For MSA, DAT loss was observed not only in the posterior putamen, but also in the ventral putamen. PSP was characterized by extensive DAT loss in both the putamen and caudate nuclei. For ^18^F-FDG PET and early phase ^18^F-FP-CIT PET, visual interpretation was based on the metabolic pattern or brain activity of the cerebral or cerebellar cortex and subcortical gray matter as published previously^13,28^. In short, the pattern for IPD was characterized by normal striatal metabolism or hypermetabolism in the bilateral dorsolateral putamen, MSA was characterized by hypometabolism in the basal ganglia and/or in the cerebellar cortex, PSP was characterized by hypometabolism in the medial frontal cortices and midbrain, while the others were classified as non-degenerative parkinsonism. MSA was further divided into cerebellar type (hypometabolism in cerebellar cortex and not in the basal ganglia), mixed type (hypometabolism in both the cerebellar cortex and basal ganglia), or parkinsonian type (hypometabolism in the basal ganglia and not in the cerebellar cortex).

### Statistical analyses

Descriptive statistics included mean ± standard deviation or frequencies for each clinical characteristic. The chi-squared test was used for categorical variables, and a one-way analysis of variance was used for quantitative variables (one-way ANOVA) with a normal distribution, and nonparametric tests (Kruskal–Wallis test) for quantitative variables without a normal distribution. P-values were based on two-sided tests and were considered statistically significant if P < 0.05 for global comparison, and P < 0.05/n for subgroup analyses to take into account the multiple comparisons. The confidence interval was be calculated based on an asymptotic variance estimate. Inter-observer agreement of the visual assessment results was assessed on the subject level using kappa values across two blinded readers. Analyses were performed using SPSS software (Statistical Package for the Social Science, version 21.0, Chicago, IL, USA).

### Ethics approval

All procedures performed in studies involving human participants were in accordance with the ethical standards of the institutional research committee and with the principles of the1964 Declaration of Helsinki and its later amendments.

## Results

### Subjects

The demographics and clinical characteristics of the subjects are summarized in Table 1. Among the 141 subjects, the average age was 61.7 ± 9.3 years, and 45.4% of the subjects were male. The mean duration of symptoms to ^18^F-FP-CIT PET was 2.0 ± 1.4 years, while the mean duration from the initial visit to ^18^F-FP-CIT PET was 0.6 ± 1.0 years. The mean clinical follow-up after ^18^F-FP-CIT PET was 2.8 ± 2.4 years, and the mean scanning interval between ^18^F-FP-CIT and ^18^F-FDG PET was 7.5 ± 82.0 days. Among patients with IPD, the mean Hoehn and Yahr stage was 2.2 ± 0.7, and the mean UPDRS III scores were 17.6 ± 9.4.

**Table 1:** Demographics and clinical characteristics.

Total	Non-DP	IPD	MSA-C	MSA-P	PSP
(n = 141)	(n = 10)	(n = 36)	(n = 46)	(n = 31)	(n = 18)
Age (years)	63.9 ± 8.2	65.0 ± 8.2	57.3 ± 8.2	60.9 ± 8.4	64.5 ± 7.7
Sex (male, female)	4:6	16:20	26:20	8:23	10:8
Symptom duration at FP-CIT PET (years)	NA	2.1 ± 1.9	1.8 ± 1.3	2.4 ± 1.5	2.5 ± 1.4
Hoehn and Yahr stage	NA	2.2 ± 0.7	NA	NA	NA
UPDRS III	NA	17.6 ± 9.4	NA	NA	NA
Duration from initial visit to PET (years)	0.1 ± 0.1	0.9 ± 1.9	0.4 ± 0.7	0.9 ± 1.1	0.9 ± 1.0
Follow-up after FP-CIT PET (years)	2.0 ± 1.7	3.9 ± 2.8	2.3 ± 1.9	2.2 ± 2.1	3.0 ± 2.6
Scanning interval (days)	29.8 ± 84.1	37.6 ± 111.1	16.7 ± 55.4	14.9 ± 33.3	7.1 ± 161.8

*Non-DP* non-degenerative parkinsonism, *PET* positron emission tomography, *FU* follow-up, *IPD* idiopathic Parkinson’s disease, *MSA-P* multiple system atrophy, Parkinson type, *MSA-C* multiple system atrophy, cerebellar type, *PSP* progressive supranuclear palsy, *UPDRS* unified Parkinson's disease rating scale, *NA* not applicable.

### Visual assessment

Visual assessment of early phase ^18^F-FP-CIT PET, delayed phase ^18^F-FP-CIT PET, early and delayed (dual) phase ^18^F-FP-CIT PET, ^18^F-FDG PET, and ^18^F-FDG and delayed phase ^18^F-FP-CIT PET images were in agreement with the clinical diagnosis in 61.7%, 69.5%, 95.7%, 81.6%, and 97.2% of cases, respectively (Table 2). Representative PET images are shown in Fig. 1. Visual assessment of each PET scan of subjects with clinical diagnosis followed more than 1 year after PET scans also showed similar results of 57.3%, 68.0%, 96.1%, 78.6%, and 97.1% of cases, respectively (Table 3).

**Table 2:** Classification matrix of visual assessment of PET scans compared to clinical diagnosis.

PET	Visual assessment	Clinical diagnosis			
		Non-DP	IPD	MSA	PSP
		(n = 10)	(n = 36)	(n = 77)	(n = 18)
Early FPCIT	Non-DP	9 (90.0%)	31 (86.1%)	5 (6.5%)	8 (44.4%)
	IPD	0 (0.0%)	3 (8.3%)	1 (1.3%)	2 (11.1%)
	MSA	1 (10.0%)	1 (2.8%)	70 (90.9%)	3 (16.7%)
	PSP	0 (0.0%)	1 (2.8%)	1 (1.3%)	5 (27.8%)
Delayed FPCIT	Non-DP	10 (100.0%)	1 (2.8%)	28 (36.4%)	0 (0.0%)
	IPD	0 (0.0%)	34 (94.4%)	9 (11.7%)	0 (0.0%)
	MSA	0 (0.0%)	0 (0.0%)	38 (49.4%)	2 (11.1%)
	PSP	0 (0.0%)	1 (2.8%)	2 (2.6%)	16 (88.9%)
Dual FPCIT	Non-DP	9 (90.0%)	2 (5.6%)	2 (2.6%)	0 (0.0%)
	IPD	0 (0.0%)	33 (91.7%)	0 (0.0%)	2 (11.1%)
	MSA	1 (10.0%)	0 (0.0%)	77 (100.0%)	0 (0.0%)
	PSP	0 (0.0%)	1 (2.8%)	0 (0.0%)	16 (88.9%)
FDG	Non-DP	9 (90.0%)	17 (47.2%)	1 (1.3%)	0 (0.0%)
	IPD	1 (10.0%)	12 (33.3%)	0 (0.0%)	0 (0.0%)
	MSA	0 (0.0%)	6 (16.7%)	76 (98.7%)	0 (0.0%)
	PSP	0 (0.0%)	1 (2.8%)	0 (0.0%)	18 (100.0%)
FDG + delayed FPCIT	Non-DP	10 (100.0%)	2 (5.6%)	0 (0.0%)	0 (0.0%)
	IPD	0 (0.0%)	33 (91.7%)	0 (0.0%)	0 (0.0%)
	MSA	0 (0.0%)	0 (0.0%)	77 (100.0%)	0 (0.0%)
	PSP	0 (0.0%)	1 (2.8%)	0 (0.0%)	17 (94.4%)

**Table 3:** Classification matrix of visual assessment of PET scans compared to clinical diagnosis in subjects followed for more than 1 year after PET scan.

PET	Visual assessment	Clinical diagnosis			
		Non-DP	IPD	MSA	PSP
		(n = 6)	(n = 31)	(n = 52)	(n = 14)
Early FPCIT	NC	6 (100.0%)	26 (83.9%)	5 (9.6%)	8 (57.1%)
	IPD	0 (0.0%)	3 (9.7%)	1 (1.9%)	1 (7.1%)
	MSA	0 (0.0%)	1 (3.2%)	45 (86.5%)	0 (0.0%)
	PSP	0 (0.0%)	1 (3.2%)	1 (1.9%)	5 (35.7%)
Delayed FPCIT	NC	6 (100.0%)	1 (3.2%)	20 (38.5%)	0 (0.0%)
	IPD	0 (0.0%)	30 (96.8%)	8 (15.4%)	0 (0.0%)
	MSA	0 (0.0%)	0 (0.0%)	22 (42.3%)	2 (14.3%)
	PSP	0 (0.0%)	0 (0.0%)	2 (3.8%)	12 (85.7%)
Dual FPCIT	NC	6 (100.0%)	2 (6.5%)	0 (0.0%)	0 (0.0%)
	IPD	0 (0.0%)	29 (93.5%)	0 (0.0%)	2 (14.3%)
	MSA	0 (0.0%)	0 (0.0%)	52 (100.0%)	0 (0.0%)
	PSP	0 (0.0%)	0 (0.0%)	0 (0.0%)	12 (85.7%)
FDG	NC	5 (83.3%)	14 (45.2%)	1 (1.9%)	0 (0.0%)
	IPD	1 (16.7%)	11 (35.5%)	0 (0.0%)	0 (0.0%)
	MSA	0 (0.0%)	6 (19.4%)	51 (98.1%)	0 (0.0%)
	PSP	0 (0.0%)	0 (0.0%)	0 (0.0%)	14 (100.0%)
FDG + delayed FPCIT	NC	6 (100.0%)	2 (6.5%)	0 (0.0%)	0 (0.0%)
	IPD	0 (0.0%)	29 (93.5%)	0 (0.0%)	1 (7.1%)
	MSA	0 (0.0%)	3 (9.7%)	52 (100.0%)	0 (0.0%)
	PSP	0 (0.0%)	0 (0.0%)	0 (0.0%)	13 (92.9%)

Early phase ^18^F-FP-CIT PET images showed 90.0% and 90.9% concordance with clinical diagnosis in subjects with non-DP and MSA, respectively, and 8.3% and 27.8% in subjects with IPD and PSP, respectively. Delayed phase ^18^F-FP-CIT PET images showed 100.0%, 94.4%, and 88.9% concordance in subjects with non-DP, IPD, and PSP, and 49.4% concordance with subjects with MSA. Dual phase ^18^F-FP-CIT PET images showed similar or higher than 90% concordance in all groups. ^18^F-FDG PET images showed equal or greater than 90% concordance in subjects with non-DP, MSA, and PSP, but only 33.3% concordance in subjects with IPD. The combination of ^18^F-FDG and delayed phase ^18^F-FP-CIT PET images yielded over 90% concordance in all groups.

Visual assessment of early phase ^18^F-FP-CIT PET showed 88.9% and 97.2% concordance with those of ^18^F-FDG PET in subjects with non-DP and MSA-C, 41.7–61.1% concordance in imaging subtypes of MSA in subjects with MSA-P, and 27.8% concordance in subjects with PSP. Delayed phase ^18^F-FP-CIT PET showed 100.0% and 88.9% concordance with ^18^F-FDG PET and clinical diagnosis in subjects with non-DP and PSP, 58.7–77.8% concordance in imaging subtypes of MSA in subjects with MSA-P, and 25.0–88.9% concordance in concordance in imaging subtypes of MSA in subjects with MSA-C. In subjects with IPD, it showed 94.4% concordance with delayed phase ^18^F-FP-CIT PET, but 33.3% concordance with ^18^F-FDG PET (Table 4).

**Table 4:** Classification matrix of visual assessment of early and delayed phase FP-CIT PET compared to FDG PET according to clinical diagnosis.

Clinical diagnosis		Non-DP		IPD					MSA-C			MSA-P			PSP
		(n = 10)		(n = 36)					(n = 46)			(n = 31)			(n = 18)
FDG		Non-DP (n = 9)	IPD (n = 1)	Non-DP (n = 17)	IPD (n = 12)	MSA-C (n = 3)	MSA-P (n = 3)	PSP (n = 1)	Non-DP (n = 1)	MSA-C (n = 36)	MSA-M (n = 9)	MSA-C (n = 1)	MSA-M (n = 18)	MSA-P (n = 12)	PSP (n = 18)
Early FP-CIT	Non-DP	8 (88.9%)	1 (100.0%)	16 (94.1%)	10 (83.3%)	2 (66.7%)	2 (66.7%)	1 (100.0%)	0 (0.0%)	1 (2.8%)	0 (0.0%)	0 (0.0%)	0 (0.0%)	4 (33.3%)	8 (44.4%)
	IPD	0 (0.0%)	0 (0.0%)	0 (0.0%)	2 (16.7%)	0 (0.0%)	1 (33.3%)	0 (0.0%)	0 (0.0%)	0 (0.0%)	0 (0.0%)	0 (0.0%)	1 (6.7%)	0 (0.0%)	2 (11.1%)
	MSA-C	1 (11.1%)	0 (0.0%)	0 (0.0%)	0 (0.0%)	1 (33.3%)	0 (0.0%)	0 (0.0%)	1 (100.0%)	35 (97.2%)	2 (22.2%)	1 (50.0%)	1 (6.7%)	0 (0.0%)	2 (11.1%)
	MSA-M	0 (0.0%)	0 (0.0%)	0 (0.0%)	0 (0.0%)	0 (0.0%)	0 (0.0%)	0 (0.0%)	0 (0.0%)	0 (0.0%)	7 (77.8%)	0 (0.0%)	11 (61.1%)	2 (14.2%)	0 (0.0%)
	MSA-P	0 (0.0%)	0 (0.0%)	0 (0.0%)	0 (0.0%)	0 (0.0%)	0 (0.0%)	0 (0.0%)	0 (0.0%)	0 (0.0%)	0 (0.0%)	0 (0.0%)	5 (27.8%)	5 (41.7%)	1 (5.6%)
	PSP	0 (0.0%)	0 (0.0%)	1 (5.7%)	0 (0.0%)	0 (0.0%)	0 (0.0%)	0 (0.0%)	0 (0.0%)	0 (0.0%)	0 (0.0%)	0 (0.0%)	0 (0.0%)	1 (7.1%)	5 (27.8%)
Delayed FP-CIT	Non-DP	9 (100.0%)	1 (100%)	0 (0.0%)	0 (0.0%)	0 (0.0%)	1 (33.3%)	0 (0.0%)	1 (100.0%)	25 (69.4%)	1 (11.1%)	0 (0.0%)	1 (5.6%)	0 (0.0%)	0 (0.0%)
	IPD	0 (0.0%)	0 (0.0%)	17 (100.0%)	12 (100.0%)	3 (100%)	2 (66.7%)	0 (0.0%)	0 (0.0%)	2 (5.6%)	0 (0.0%)	1 (100.0%)	1 (5.6%)	5 (41.7%)	0 (0.0%)
	MSA	0 (0.0%)	0 (0.0%)	0 (0.0%)	0 (0.0%)	0 (0.0%)	0 (0.0%)	0 (0.0%)	0 (0.0%)	9 (25.0%)	8 (88.9%)	0 (0.0%)	14 (77.8%)	7 (58.3%)	2 (11.1%)
	PSP	0 (0.0%)	0 (0.0%)	0 (0.0%)	0 (0.0%)	0 (0.0%)	0 (0.0%)	1 (100.0%)	0 (0.0%)	0 (0.0%)	0 (0.0%)	0 (0.0%)	2 (11.1%)	0 (0.0%)	16 (88.9%)

In visual assessment of each PET scan, the κ value of the two raters for early phase ^18^F-FP-CIT PET, delayed phase ^18^F-FP-CIT PET, early and delayed (dual) phase ^18^F-FP-CIT PET, ^18^F-FDG PET, and ^18^F-FDG and delayed phase ^18^F-FP-CIT PET images were 0.94, 0.76, 0.87, 0.64, and 0.90 respectively.

## Discussion

In this study, we evaluated the diagnostic performance of dual phase ^18^F-FP-CIT PET for the detection and differentiation of degenerative parkinsonism. To this end, we compared early phase ^18^F-FP-CIT PET and delayed phase ^18^F-FP-CIT PET with clinical diagnosis or ^18^F-FDG PET, either respectively or in combination. Our results showed that early phase ^18^F-FP-CIT PET can differentiate MSA from other types of DP, and that delayed phase ^18^F-FP-CIT PET can not only detect DP from non-DP, but also differentiate PSP from others. Overall, our findings indicated that dual phase ^18^F-FP-CIT PET can provide equivalent information on overall functional activity and DAT availability compared to a combination of ^18^F-FDG PET and delayed phase ^18^F-FP-CIT PET for the differential diagnosis of parkinsonian disorders with a single injection.

Tracers with high lipophilicity may have a higher first-pass influx rate (K1); therefore, early phase ^18^F-FP-CIT PET imaging can provide a measure of the rate of ligand influx into the brain, K1^20^. Regional K1 is highly correlated with regional cerebral blood flow. As blood flow is coupled tightly to metabolism, early phase ^18^F-FP-CIT PET can provide similar diagnostic information to ^18^F-FDG PET. Typical pathologic patterns in the overall brain functional activity that show neuronal dysfunction and degeneration, could be recognized for different parkinsonian disorders in both images as we have previously reported ^25^.

However, diagnostic performance of early phase ^18^F-FP-CIT PET is variable among groups. It correctly classified MSA but not in PSP which is different from ^18^F-FDG PET. Furthermore, the characteristic metabolic pattern of PSP is hypometabolism on the medial frontal cortex and midbrain. Early or perfusion phase imaging usually underestimates cortical perfusion than that of cerebellum compared to the metabolism^22^. This leads to a smaller regional difference in cortical perfusion, which is less than that of the metabolism; this represents a hurdle for the detection of hypoperfusion in the medial frontal cortex on early phase images in subjects with PSP. In dynamic imaging of ^18^F-FP-CIT PET, the time activity curve showed moderate uptake in the midbrain in subjects with IPD, which reflected serotonergic binding in these regions^29^. These findings suggest that activities of the midbrain can affect both blood flow and serotonergic binding that can interfere with the detection of hypoperfusion of the midbrain in subjects with PSP. Furthermore, the low signal to noise ratio of early phase images could be disadvantageous to evaluate the perfusion of small lesion such as those in the midbrain^24,28^.

Similarly, delayed phase ^18^F-FP-CIT PET can discriminate IPD and PSP from other types of parkinsonism well, but not in MSA. Over two thirds of the subjects with MSA-C, clinically and metabolically, showed a visually normal DAT binding pattern on delayed image. However, severe DAT loss was observed both in the putamen and caudate in subjects with PSP, which is consistent with previous reports^8,9^.

A clinical implementation of dual phase ^18^F-FP-CIT PET for the differential diagnosis of parkinsonian disorders has several advantages over a combination of ^18^F-FDG PET and delayed phase ^18^F-FP-CIT PET for reducing the radiation dose, and saving time and cost to the patients. When only using a single ^18^F-FDG PET scan, the radiation dose is reduced by approximately 70% from about 10 mSv for the combination of ^18^F-FDG PET and delayed phase ^18^F-FP-CIT PET to approximately 2.7 mSv for ^18^F-FP-CIT PET^30^. From a patient perspective, a single scan is more convenient and inexpensive than a dual examination. Furthermore, there is no need for fasting several hours before scanning, and a quiet and dimly lit waiting room is provided for optimal glucose utilization in the brain. Finally, the acquisition of early phase ^18^F-FP-CIT PET images only takes 10 min and there is no need for processing on the contrary to generate the parametric relative delivery image (R1). The R1 image requires dynamic acquisition for more than 2 h, which certainly increases the risk for significant movement. However, the clinical utilization can be impaired since the majority of candidates for ^18^F-FP-CIT PET have parkinsonism and cannot cooperate for such a long time in the same position. This could also be problematic for the PET facility due to the longer time spent occupying the scanner.

The present study was subject to some limitations. First, the composition of the study population in terms of the different types of parkinsonian disorders was not representative of the usual prevalence of each disease, which is over 70% for IPD, followed by MSA and PSP. Subjects with IPD or non-DP generally do not undergo both ^18^F-FP-CIT PET and ^18^F-FDG PET. Differential diagnosis of parkinsonism is performed at specialized hospitals, which often have a high prevalence of atypical parkinsonism, including MSA or PSP. Second, the use of clinical diagnosis as a gold standard may be problematic. As mentioned before, diagnosing PD correctly based on clinical symptoms can be difficult, and misdiagnosis occurs in up to 25% of patients in general practice. We have tried to overcome this limitation by using movement disorder specialists for selecting the participants. Indeed, the diagnostic performance was consistent in the subgroup of the study population followed up for more than 1 year. Finally, we did not perform quantitative analysis, which would have provided valuable information about the degree of DAT loss, especially in patients with MSA-C with a visually normal DAT binding pattern on delayed ^18^F-FP-CIT PET. However, these additional analyses require more time and effort and are not readily available in routine clinical practice.

In summary, dual phase ^18^F-FP-CIT PET may represent a powerful alternative to the combination of ^18^F-FDG PET and delayed ^18^F-FP-CIT PET for the detection and differential diagnosis of parkinsonian disorders.

## Data Availability

The datasets generated during and/or analysed during the current study are available from the corresponding author on reasonable request.
